# Physicochemical Characteristics of Amphipathic Peptides and Their Cytotoxic Effects on Cancer and Normal Cell Lines

**DOI:** 10.3390/ijms27072952

**Published:** 2026-03-24

**Authors:** Iwona Golonka, Katarzyna E. Greber, Zofia Łapińska, Dariusz Wyrzykowski, Krzysztof Żamojć, Emilia Sikorska, Julita Kulbacka, Wiesław Sawicki, Witold Musiał

**Affiliations:** 1 Department of Physical Chemistry and Biophysics, Faculty of Pharmacy, Wroclaw Medical University, Borowska 211A, 50-556 Wroclaw, Poland; iwona.golonka@umw.edu.pl; 2 Department of Physical Chemistry, Faculty of Pharmacy, Medical University of Gdańsk, Al. Gen. J. Hallera 107, 80-416 Gdańsk, Poland; greber@gumed.edu.pl (K.E.G.); wsawicki@gumed.edu.pl (W.S.); 3 Department of Chemistry, Molecular and Cellular Biology, Faculty of Pharmacy, Wroclaw Medical University, Borowska 211A, 50-556 Wroclaw, Poland; zofia.lapinska@umw.edu.pl (Z.Ł.); julita.kulbacka@umw.edu.pl (J.K.); 4 Department of Immunology and Bioelectrochemistry, National Research Institute of Innovative Medicine, LT-08406 Vilnius, Lithuania; 5 Department of General and Inorganic Chemistry, Faculty of Chemistry, University of Gdańsk, Wita Stwosza 63, 80-308 Gdańsk, Poland; dariusz.wyrzykowski@ug.edu.pl (D.W.); krzysztof.zamojc@ug.edu.pl (K.Ż.); 6 Laboratory of Structural Research of Biopolymers, Faculty of Chemistry, University of Gdańsk, Wita Stwosza 63, 80-308 Gdańsk, Poland; emilia.sikorska@ug.edu.pl

**Keywords:** peptides, surface tension, osmolality, cytotoxicity, isothermal titration calorimetry, liposome, fluorescence spectroscopy, confocal microscopy

## Abstract

The aim of this study was to investigate which physicochemical and structural properties of cationic peptides P1–P6 may determine their selective anticancer activity against melanoma cells and their interactions with tumor cell membranes. An integrated approach was applied, including characterization in solution (osmotic pressure, NaCl stability, surface tension); cytotoxicity evaluation against Me45, B16F10, and HaCaT cells; analysis of interactions with phosphatidylglycerol (POPG) model membranes using isothermal titration calorimetry and steady-state fluorescence spectroscopy; membrane permeability assays; and F-actin staining. Anticancer activity depended on positively charged residues, hydrophobic amino acids, and sequence arrangement. Tryptophan-rich peptides P2 and P5 exhibited strong membrane interactions and high efficacy after 72 h. Highly hydrophobic P4, containing long C_12_ chains with a relatively low net charge, caused nonselective lysis. P3 showed reduced activity due to insufficient amphipathicity, whereas P6, with excessive WWW and KKKK motifs, exhibited weak or nonselective effects. Thermodynamic and fluorescence analyses indicated that P2 and P5 initially bind POPG membranes via entropy-driven electrostatic interactions, followed by hydrophobic insertion of tryptophan residues, evidenced by increased fluorescence intensity and a blue shift of the emission maximum. P2, P4, and P5 induced actin cytoskeleton reorganization and increased membrane permeability, emphasizing the role of balanced amphipathicity and charge–hydrophobicity in designing selective anticancer peptides.

## 1. Introduction

In recent years, cationic amphipathic peptides, often derived from the class of antimicrobial peptides or designed de novo, have gained significant interest as potential anticancer molecules (anticancer peptides, ACPs) [1,2,3]. Compared to traditional cancer therapies, they demonstrate relatively high cellular penetration and potentially selective action against cancer cells in a short time [4,5,6]. The mechanism of action of ACPs is largely related to differences in the composition of cell membranes of cancer cells compared to healthy cells, including increased expression of negatively charged phospholipids (e.g., phosphatidylserine) on the outer layer of the membrane, altered membrane fluidity, and different pH in the tumor microenvironment [7,8,9,10]. The positive charge of peptides promotes their electrostatic attraction to the surface of cancer cells, while hydrophobic/amphipathic properties enable interaction with the lipid layer, often leading to membrane destabilization, penetration into the cell interior, and initiation of apoptotic pathways [11,12,13]. Despite numerous studies on ACPs, achieving high selectivity—that is, effective destruction of cancer cells with minimal damage to healthy cells—remains a key challenge. The literature emphasizes that increasing attention should be paid not only to the peptide sequence but also to its physicochemical and thermodynamic properties, such as net charge, molecular weight, hydrophobicity/amphipathicity, aggregation tendency, thermal stability, surface activity, and solution parameters (e.g., surface tension, osmotic pressure) [14,15,16]. However, it should be remembered that too high a net charge or excessive hydrophobicity may increase toxicity to healthy cells and promote aggregation of the peptide in solution [17,18]. To address these challenges, increasing emphasis has been placed on the use of biophysical methods that enable detailed characterization of peptide–membrane interactions. Among them, isothermal titration calorimetry (ITC) provides direct insight into the binding affinity and thermodynamic drivers governing peptide–lipid complex formation [19,20,21]. Studies on cationic amphipathic peptides interacting with anionic lipids such as phosphatidylglycerol (POPG), a component commonly enriched on the surfaces of cancer cells, reveal that binding often exhibits a substantial entropic contribution, indicating a major role of hydrophobic interactions in addition to electrostatics [22,23]. Aromatic residues, especially tryptophan, have been shown to significantly enhance the stabilization of peptide–lipid complexes through insertion into hydrophobic regions of the membrane [24,25,26]. Additional insights into the mechanism of ACP action can be obtained from fluorescence spectroscopy. The observed blue shift of tryptophan emission indicates its relocation to a more hydrophobic environment within or near the lipid bilayer, confirming direct and specific interactions of ACPs with anionic lipids and the contribution of hydrophobic insertion [27,28,29]. Cellular models are often proposed for detailed evaluation of the potential cytotoxicity of newly developed bioactive substances, with several standard cell lines, including HeLa cell lines [30] and others that have been applied to peptides [31].

In this work, we analyzed six cationic peptides (designated P1–P6) whose physicochemical and microbiological properties were investigated in previous studies for their activity against *Cutibacterium acnes* [32,33,34] and effects on model lipid monolayers [35,36,37]. The current study focused on elucidating the relationship between these properties and the peptides’ anticancer activity. A multi-step research approach was employed: (i) characterization of the physicochemical and thermodynamic properties of the peptides in solution, including osmotic pressure profiles, surface tension behavior upon dilution, and stability in NaCl solution, to evaluate their suitability for cellular studies; (ii) assessment of peptide cytotoxicity against a human melanoma cell line (Me45), a murine melanoma cell line (B16F10), and normal keratinocytes (HaCaT) after 24 and 72 h of incubation. Peptide–lipid interactions were further investigated using isothermal titration calorimetry (ITC) and fluorescence spectroscopy. The objective was to correlate peptide structural characteristics with their selective cytotoxicity toward cancer cells. This study was designed as an initial screening to identify the most promising peptide candidates. To better understand the mechanism of action and selectivity of cationic peptides, a qualitative assessment of their effects on cell morphology, actin cytoskeleton organization, and membrane permeability in melanoma cell lines was performed.

## 2. Results

### 2.1. Physicochemical and Thermodynamic Profiling of Peptides in Solution

#### 2.1.1. Peptide Stability at 0.125 mg/mL in 0.9% NaCl Solution

The stability of six peptides (P1–P6, 0.125 mg/mL) in NaCl solution was monitored over time (24–168 h) at the maximum wavelength for each peptide (204–280 nm) using UV absorbance measurements (Table S1). The results showed relatively constant absorbance, suggesting good chemical stability of the peptides. Maintaining stability in solution is important for the reproducibility of in vitro experiments and further biological and therapeutic studies.

#### 2.1.2. Osmotic Properties

The osmotic properties and thermal behavior of six cationic peptides (P1–P6) were investigated over a concentration range of 0.0625–0.125 mg/mL (Table 1). Osmotic pressure increased with peptide concentration for all samples. P5 showed the highest values (289–315 mOsm/kg), P6 the lowest (275–280 mOsm/kg), and P2 and P4 exhibited intermediate, nearly linear increases. The temperature decreased slightly with increasing concentration, with the largest decrease observed for P5 (from −0.536 to −0.585 °C) and the smallest for P6 (from −0.510 to −0.520 °C).

#### 2.1.3. Analysis of Surface Tension of Peptides (P1–P6) as a Function of Concentration

**Figure 1:**
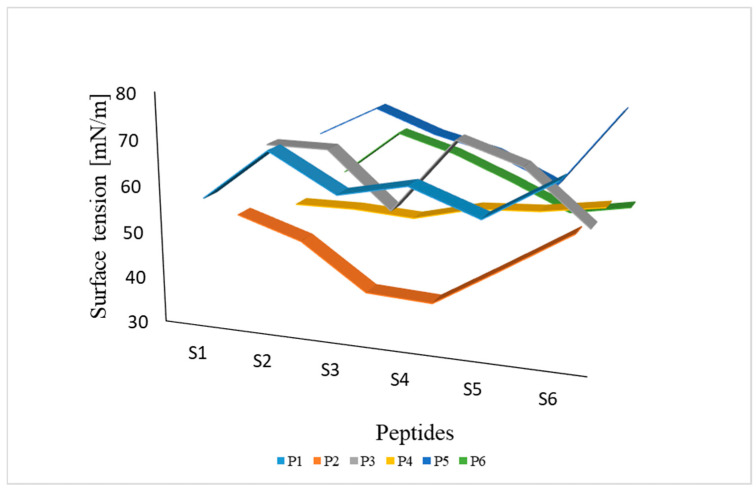
Surface tension [mN/m] of six cationic amphipathic peptides (P1–P6) at selected concentrations in aqueous solution (concentration [mmol/L]: S1—0.0024, S2—0.0071, S3—0.0214, S4—0.0641, S5—0.1922, S6—0.5765).

The surface tension of peptide P1 remained relatively stable at approximately 56.87 to 68.36 mN/m at various concentrations (Figure 1). Peptide P2 induced a decrease in surface tension to approximately 34.44 mN/m at low concentrations, followed by a subsequent increase. This behavior may indicate the attainment of the critical micelle concentration (CMC), which could be estimated to be around 0.0427 mmol/L. Surface tension values for peptide P3 were relatively high and variable, most often above 49.08 mN/m, with distinct peaks reaching 66 mN/m. Peptide P4 was studied over a wider range of higher concentrations. Its surface tension was lower, approximately 44–53 mN/m, and increased slightly with decreasing concentration. A CMC at 0.8511 mmol/L concentration was unambiguously determined for this peptide. The surface tension of peptide P5 exhibited fluctuations, remaining between 50.17 and 69.33 mN/m. Similar to P1, this peptide was characterized by relatively stable surface activity over a wide range of concentrations. Peptide P6, on the other hand, exhibited more pronounced surface tension fluctuations, ranging from approximately 39.67 to 56.86 mN/m.

The results indicate that for each peptide, there were differences in the mean surface tension values between the solutions. Analysis of variance (ANOVA) was performed at a 95% confidence level. To determine which solutions differed from each other, a Tukey post-hoc test was applied. In most cases, all solutions of a given peptide differed significantly from each other, except for the lack of significant differences between S1 (0.0024 mmol/L) and S2 (0.0071 mmol/L) as well as S3 (0.0214 mmol/L) and S6 (0.5765 mmol/L) for peptide P3.

### 2.2. Cell Viability of Human Melanoma (Me45), Mouse Melanoma (B16F10), and Normal Human Keratinocytes (HaCaT) After 24 h and 72 h Incubation with Peptides P1–P6

In cytotoxicity analyses performed with the MTT assay, the viability of human melanoma Me45, murine melanoma B16F10, and human keratinocyte HaCaT cells was evaluated after 24 and 72 h incubation with peptides P1–P6. The results revealed marked differences in the activity of individual peptides with respect to both potency and selectivity. The cytotoxic activity of peptides P1–P6 was additionally evaluated by determining IC_50_ values based on the MTT assay results (Table 2). The results demonstrate substantial variability in peptide potency depending on both the cell line and exposure duration. Moreover, selectivity toward melanoma cells was further quantified by calculating the selectivity index (SI) and, where applicable, the minimal selectivity index (SI-min), the values of which are presented in Table S2 and form the basis for the assessment of selective activity.

**Figure 2:**
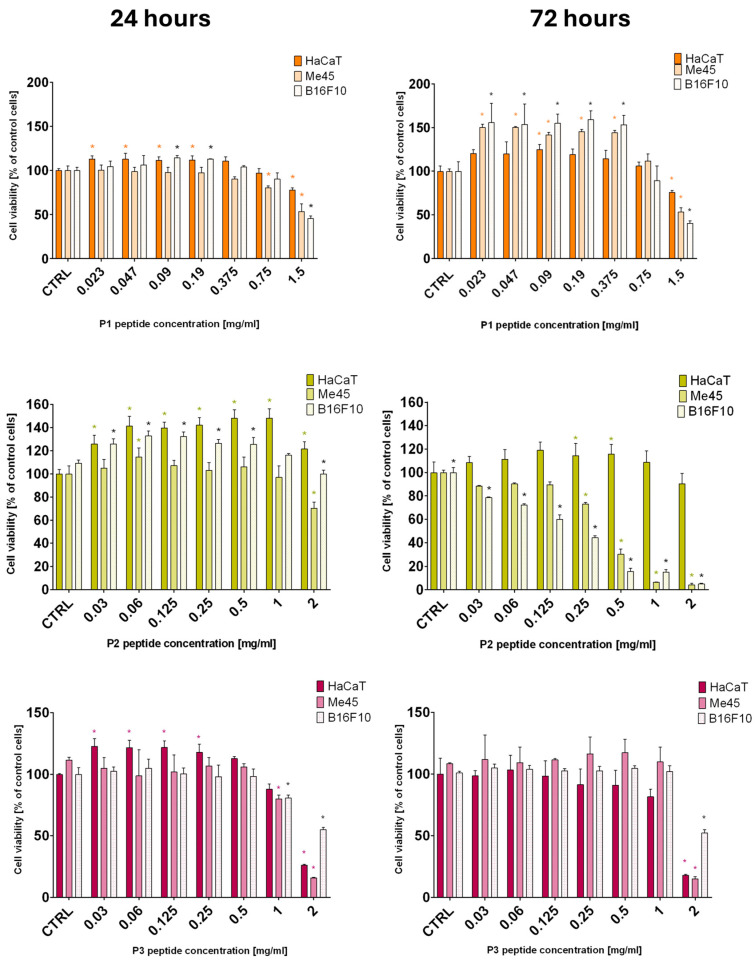
Cell viability of human melanoma (Me45), mouse melanoma (B16F10), and normal human keratinocytes (HaCaT) after 24 h (**left**) and 72 h (**right**) incubation with peptides P1–P3. Notes: mean ± SD, *n*= 3. * *p* < 0.05 compared to control (CTRL, 0 mg/mL).

Peptide P1 (Figure 2) displayed limited cytotoxic activity. After 24 h at the highest concentration tested (1.5 mg/mL), the viability of Me45 and B16F10 cells decreased to ~53% and ~46%, respectively, while HaCaT cells remained at ~78%. Lower concentrations did not produce a significant reduction in cell viability. Notably, a prolonged incubation (72 h) revealed a potential stimulatory effect on proliferation or metabolic activity at concentrations ≤0.375 mg/mL, more pronounced in tumor cells than in normal keratinocytes. Only at the highest concentration (1.5 mg/mL) did P1 exert a marked cytotoxic effect after 72 h (Me45 ~53%, B16F10 ~41%, HaCaT ~76%; all statistically significant). These results indicate moderate antitumor activity of P1 at high concentrations, with relatively limited impact on normal cells. Peptide P2 exhibited a different profile. After 24 h, no significant reduction in viability was observed in any cell line (except for Me45 at 2 mg/mL, ~70%). In contrast, after 72 h, P2 displayed strong, dose-dependent cytotoxicity toward melanoma cells, while maintaining high HaCaT viability. From 0.25 mg/mL, a significant decrease in Me45 and B16F10 viability was observed, with minimal effect on HaCaT. For instance, at 0.5 mg/mL after 72 h, Me45 viability was ~31%, B16F10 was ~16%, while HaCaT viability remained ~116% relative to the control. These findings suggest delayed but selective cytotoxic activity of P2. Peptide P3 showed limited and non-selective cytotoxicity. At concentrations <2 mg/mL, no significant reduction in viability was noted, and values above the control were often recorded in tumor cell lines. Only the maximal dose (2 mg/mL) resulted in pronounced cytotoxicity in all cell lines, including HaCaT. After 72 h, viability was reduced to ~18% (HaCaT), ~15% (Me45), and ~52% (B16F10), indicating a lack of selective antitumor activity. Values exceeding 100% reflected an increase in metabolic activity relative to untreated control cells (CTRL; set as 100%) and could indicate stimulation of mitochondrial dehydrogenase activity or mild proliferative effects induced by low concentrations of the tested compounds [38].

**Figure 3:**
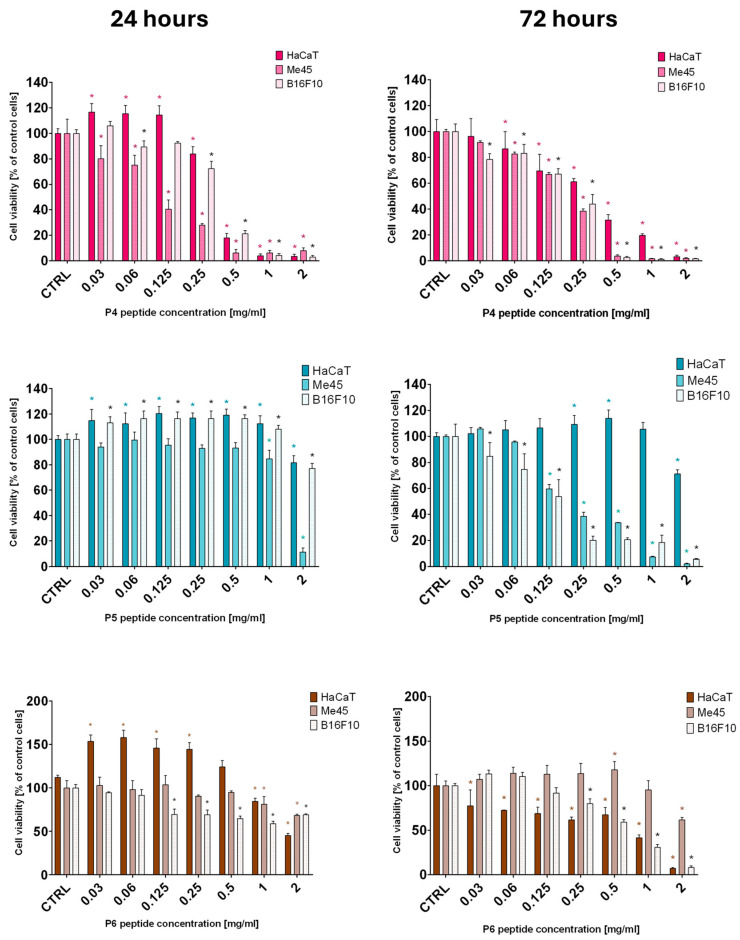
Cell viability of human melanoma (Me45), mouse melanoma (B16F10), and normal human keratinocytes (HaCaT) after 24 h (**left**) and 72 h (**right**) incubation with peptides P4–P6. Notes: mean ± SD, *n* = 3. * *p* < 0.05 compared to control (CTRL, 0 mg/mL).

Peptide P4 (Figure 3) was strongly cytotoxic at concentrations ≥0.5 mg/mL, rapidly reducing melanoma cell viability but also affecting HaCaT cells. At higher concentrations, nearly complete loss of viability was observed across all cell lines, irrespective of incubation time, indicating potency. Peptide P5 revealed cytotoxic potential primarily after prolonged exposure, with relative selectivity toward tumor cells. After 24 h, no significant effects were detected except for Me45 at 2 mg/mL, where viability dropped to ~11% (HaCaT ~82%, B16F10 ~77%). After 72 h, a clear, dose-dependent effect was observed: from 0.25 mg/mL, viability of Me45 and B16F10 decreased substantially, while HaCaT cells remained unaffected or slightly above control levels (~110%). At the highest concentrations (1–2 mg/mL), melanoma cell viability dropped to single-digit values, whereas HaCaT viability was only partially reduced. These results suggest selective antitumor activity of P5 after 72 h of incubation. Peptide P6 showed no significant antitumor activity, with data suggesting higher toxicity toward normal cells. After 24 h, a moderate decrease was observed mainly in B16F10 (~60% at 1 mg/mL), while Me45 cells remained largely unaffected (~82%). At 2 mg/mL, HaCaT viability dropped to ~45%, B16F10 to ~69%, and Me45 remained at ~68%. After 72 h, the cytotoxic effect intensified primarily in HaCaT and B16F10, whereas Me45 cells remained close to control values across most concentrations, decreasing only at 2 mg/mL (~62%). Thus, P6 did not demonstrate selective cytotoxicity toward human melanoma cells and the calculated selectivity index (SI) indicated only minimal preference for murine melanoma cells over normal keratinocytes (Table S2).

In HaCaT cells, most peptides exhibited low cytotoxicity. P1, P2, and P5 did not reach 50% inhibition at the highest concentrations tested (IC_50_ > 1.5–2.0 mg/mL), indicating limited activity toward normal keratinocytes (Table 2). P3 displayed moderate cytotoxicity after 24 h (IC_50_ = 3.207 mg/mL), which markedly decreased after 72 h (IC_50_ = 20.94 mg/mL). P6 showed intermediate potency (IC_50_ ≈ 1 mg/mL), whereas P4 was the most cytotoxic peptide in HaCaT cells, with IC_50_ values of 0.3129 mg/mL and 0.4093 mg/mL after 24 h and 72 h, respectively.

The Me45 melanoma line demonstrated higher overall sensitivity to several peptides. P4 consistently exhibited the strongest cytotoxic effect (IC_50_ = 0.1231–0.2077 mg/mL), followed by P3 (IC_50_ ≈ 1.1–1.3 mg/mL). Notably, both P2 and P5 showed marked time dependence: each failed to reach IC_50_ at 24 h (P2 > 2.0 mg/mL; P5 = 5.191 mg/mL), but after 72 h, their potency increased substantially, yielding IC_50_ values of 0.3907 mg/mL (P2) and 0.1083 mg/mL (P5). P1 and P6 remained largely inactive in this line (IC_50_ > 1.5–2.0 mg/mL).

The B16F10 melanoma line was the least responsive at 24 h, when only P1 (IC_50_ = 1.374 mg/mL) and P4 (IC_50_ = 0.3386 mg/mL) exhibited measurable cytotoxicity, while all remaining peptides showed IC_50_ values above the tested range. After 72 h, however, cytotoxicity increased for several compounds. P5, P2, and P4 displayed the highest potency (IC_50_ = 0.1319, 0.2385, and 0.2558 mg/mL, respectively), followed by P1 (0.7152 mg/mL) and P6 (0.9135 mg/mL). P3 remained inactive at both time points (IC_50_ > 2.0 mg/mL).

For peptides that did not reduce viability below 50% at the highest tested concentration, IC_50_ values are reported as “>maximal tested concentration”, which is consistent with the viability profiles shown in Figure 2 and Figure 3.

In summary, among the peptides tested, P2 and P5 demonstrated the most promising profiles, exhibiting strong antitumor effects after prolonged exposure (72 h; Figure 2 and Figure 3), reflected by low IC_50_ values in melanoma cell lines while maintaining high viability of normal keratinocytes (Table 2). This pattern was further supported by the calculated selectivity index (SI/SI_min_) values, indicating preferential cytotoxicity toward melanoma cells (Table S2).

### 2.3. The Thermodynamics of POPG–Peptide Interactions

**Figure 4:**
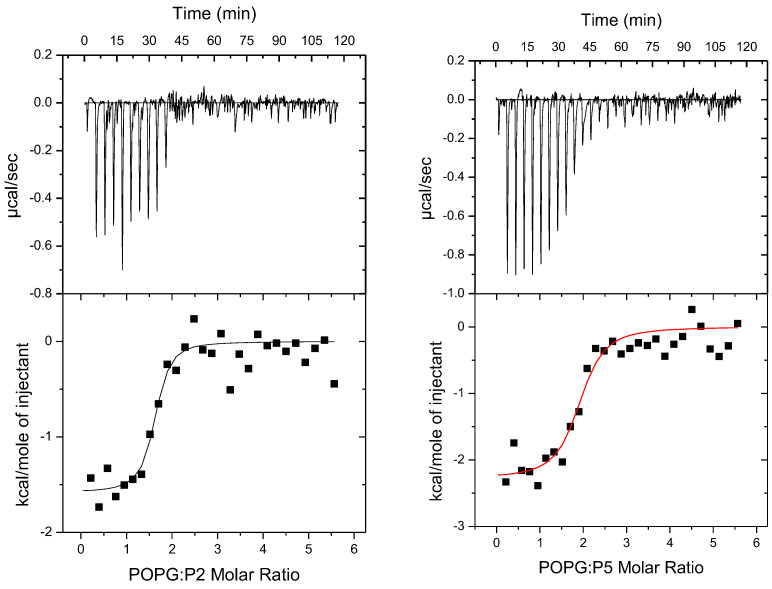
Calorimetric titration isotherms of binding interactions of the P2 and P5 peptides with POPG in a PBS buffer (pH 7.4) at 298.15 K. The upper panel shows the heat flow (μcal/sec) as a function of time, representing the rate of heat change during each injection. The lower panel shows the integrated binding isotherm.

The investigation of systems involving liposomes and peptides is often a complex and multifaceted task. Among various analytical techniques, isothermal titration calorimetry (ITC) is recognized as a particularly useful method for directly determining the fundamental thermodynamic parameters of the interactions. Specifically, ITC enables the determination of stoichiometry, binding constants, and binding enthalpy without the necessity for molecular labeling or immobilization [39,40,41]. Thus, in this study, the ITC technique was successfully applied to gain some insight into the binding interactions of POPG with the investigated peptides (P2 and P5, which showed the strongest anticancer activity among the tested peptides). Figure 4 presents representative binding isotherms for the interactions, while the parameters of these interactions are summarized in Table 3.

ITC measurements revealed that the binding of peptides P2 and P5 to the POPG lipid exhibited similar stability, as reflected in the log*K*_(ITC)_ and Δ*G*_(ITC)_ values (Table 3). This difference of approximately 0.76 kcal mol^−1^ in Δ*G*_(ITC)_ could be attributed to the higher overall positive charge of the P2 peptide (+8) compared to P5 (+4) at the experimental pH of 7.4, resulting in the stronger interaction with the anionic POPG structure. However, an assessment of the experimental errors showed that the binding affinity values (log*K*_(ITC)_) and changes in free energy (Δ*G*_(ITC)_) for both complexes remained within the experimental error range (Table 3). Therefore, it can be assumed that the thermodynamic stability of the POPG-P2 and POPG-P5 complexes was similar.

However, the nature of the binding interactions for both peptides was revealed in the balance between enthalpy (ΔH) and entropy (TΔS), which together determined the overall free energy change (ΔG). Although the interactions were slightly exothermic, ITC data showed that binding, under experimental conditions, in both cases was predominantly entropy-driven. The entropic contributions were large and favorable, while the enthalpic contributions were minor (Table 3). This profile is consistent with binding processes dominated by the hydrophobic effect and the release of ordered water upon peptide–lipid association. While an initial electrostatic attraction between the cationic peptides and the anionic POPG headgroups is a plausible first step that may position the peptides at the membrane surface, its specific energetic contribution appears to be small. The dominant thermodynamic stability instead points to subsequent hydrophobic interactions, particularly those involving tryptophan and lysine residues, as the primary driver of insertion and stable anchoring into the POPG bilayer. Such hydrophobic interactions are likely the main source of the observed entropy-driven binding, consistent with the final thermodynamic stability of the complexes.

**Figure 5:**
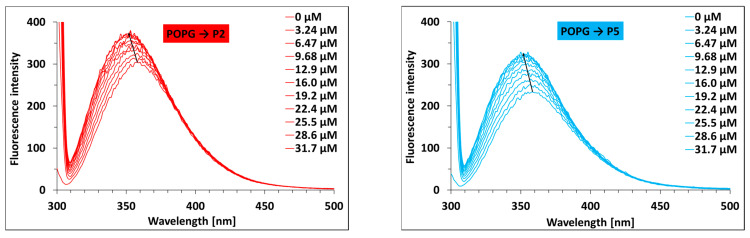
The fluorescence emission spectra of the P2 and P5 peptides with POPG. c P2/P5 = 10 µM; c POPG = 0–31.7 µM; λex = 295 nm; in a PBS buffer (pH 7.4), 298.15 K.

The assumption regarding the engagement of hydrophobic interactions is in line with the results of spectrofluorometric measurements. It has been found that for both peptides, under the interactions with POPG, an increase in fluorescence intensity and a shift (of approximately 6 nm) of the emission maximum towards shorter wavelengths (black arrows on both graphs) were observed (Figure 5). This behavior indicates a change in the hydrophobicity around the tryptophan residues, likely due to the interactions of the peptides with POPG.

### 2.4. Effect of Peptides on Actin Cytoskeleton Organization and Membrane Permeability

**Figure 6:**
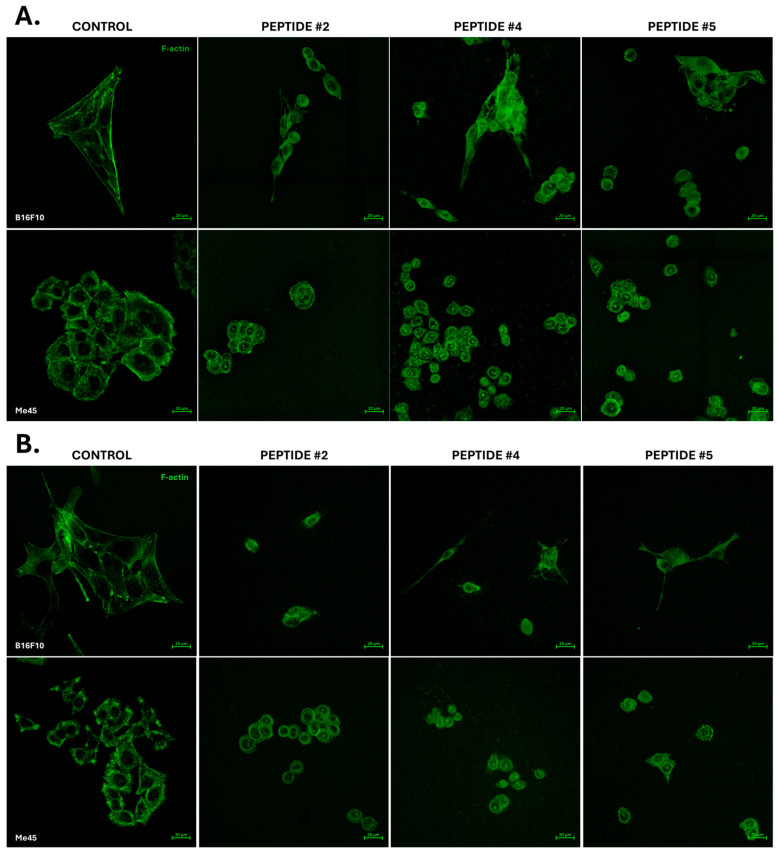
Visualization of F-actin organization in B16F10 and Me45 cells after 24 h (**A**) and 72 h (**B**) incubation with peptides: P#2 (1 mg/mL), P#4 (0.25 mg/mL), and P#5 (1 mg/mL). Cells were stained to visualize filamentous actin (F-actin) and imaged using a confocal microscope with a 40× water immersion objective. Representative images show changes in cytoskeletal organization depending on peptide treatment and incubation time. Scale bar = 20 µm.

To evaluate the effects of the investigated peptides on cellular morphology, the organization of actin filaments (F-actin) was analyzed in B16F10 and Me45 cell lines after 24 and 72 h of incubation with peptides P2, P4, and P5. Untreated cells served as the reference control (CTRL). The analysis was qualitative in nature and based on representative microscopic images (Figure 6).

Control B16F10 cells exhibited a typical spread morphology, with prominent stress fibers extending across the cytoplasm (Figure 6A). Following incubation with peptides P2 and P5, morphological alterations were observed, including cell rounding, reduced adhesion area, and decreased organization of actin filaments. The F-actin signal appeared more diffuse, and stress fibers were less distinct. In the case of peptide P4, a subset of cells retained a more spread morphology; however, a reduction in actin filament organization compared with the control was also evident. Based on qualitative image assessment, no clear differences in the extent of morphological alterations were observed between peptides P2 and P5. In Me45 cells, control conditions were characterized by the presence of distinct cell clusters with visible cortical actin. Peptide incubation resulted in morphological changes and a less organized distribution of the actin signal; however, the overall cluster structure was preserved under all analyzed conditions. Partial cell rounding and a reduction in filamentous actin organization were observed following treatment with peptides P2, P4, and P5. Based on the presented images, no definitive differences in cell number between experimental conditions were identified. In some cells, a punctate F-actin signal was observed in regions corresponding to the nuclear area.

After 72 h of incubation (Figure 6B), B16F10 cells treated with peptide P2 exhibited altered morphology and reduced actin filament organization compared with the control. Under peptide P4 treatment, cytoskeletal disorganization was also observed, without clear evidence of restoration of the typical actin architecture. Following treatment with peptide P5, cells with heterogeneous morphology and limited filament organization were present. In the Me45 cell line, morphological alterations observed at 24 h persisted after prolonged exposure. Peptide-treated cells exhibited rounding and a less organized F-actin distribution, while maintaining the presence of cell clusters. No pronounced qualitative differences in colony organization were observed between individual experimental conditions.

All tested peptides were associated with qualitatively observable reorganization of the actin cytoskeleton and alterations in cellular morphology in both cell lines. These changes primarily included a reduction in stress fiber prominence and a more diffuse distribution of the actin signal.

To complement the qualitative observations, quantitative analysis of F-actin staining was performed by measuring cell area and mean F-actin fluorescence intensity per cell.

**Figure 7:**
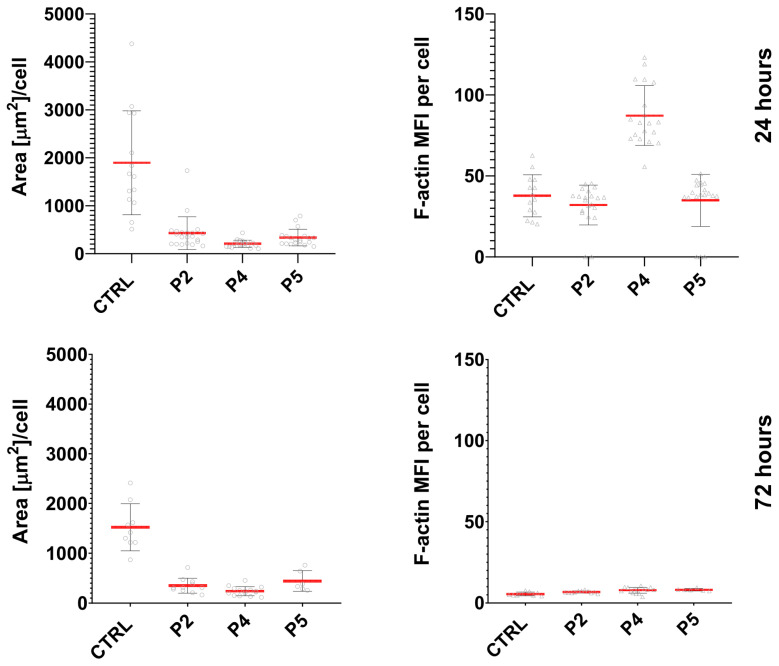
Quantitative analysis of cell area and F-actin fluorescence intensity in B16F10 cells after peptide treatment. Cells were incubated for 24 h and 72 h with peptides P2 (1 mg/mL), P4 (0.25 mg/mL), and P5 (1 mg/mL). Untreated cells served as the control (CTRL). Cell area (µm^2^/cell) and mean fluorescence intensity (MFI) of F-actin were quantified from confocal images of F-actin–stained cells. Each point represents an individual cell; red bars indicate mean ± SD.

In B16F10 cells, peptide treatment resulted in a pronounced reduction in cell area compared with control cells after both 24 h and 72 h of incubation, indicating loss of the typical spread morphology and increased cell rounding (Figure 7). This effect was observed for all tested peptides (P2, P4, and P5). Analysis of F-actin fluorescence revealed peptide-dependent changes in cytoskeletal organization. After 24 h, cells treated with peptide P4 displayed the highest mean F-actin fluorescence intensity (MFI), which, taken together with lowest area values noted for this peptide, suggests enhanced condensation or reorganization of actin filaments. P2 and P5 induced less pronounced changes. After 72 h of incubation, F-actin signal intensity decreased across all experimental groups, consistent with the loss of organized filament structures observed in the qualitative analysis.

**Figure 8:**
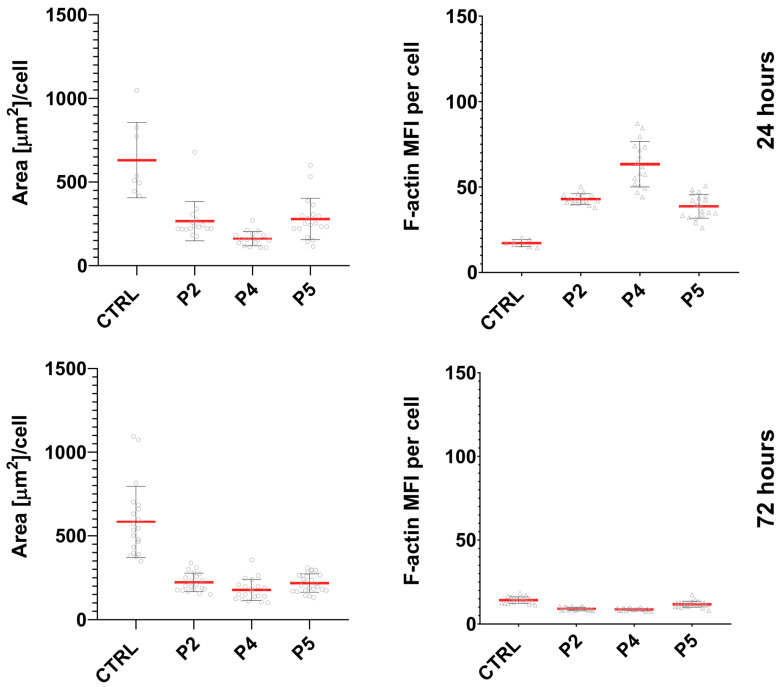
Quantitative analysis of cell area and F-actin fluorescence intensity in Me45 cells after peptide treatment. Cells were incubated for 24 h and 72 h with peptides P2 (1 mg/mL), P4 (0.25 mg/mL), and P5 (1 mg/mL). Untreated cells served as the control (CTRL). Cell area (µm^2^/cell) and mean fluorescence intensity (MFI) of F-actin were quantified from confocal images of F-actin-stained cells. Each point represents an individual cell; red bars indicate mean ± SD.

A similar trend was observed in the Me45 cell line (Figure 8). Peptide treatment was associated with a reduction in cell area compared with control cells, reflecting morphological changes consistent with partial cell rounding. Quantitative analysis of F-actin fluorescence intensity demonstrated increased actin signal after 24 h of treatment, particularly in cells exposed to peptide P4, indicating cytoskeletal reorganization. After prolonged incubation (72 h), F-actin fluorescence intensity decreased in all experimental conditions.

Taken together, the quantitative analysis supports the qualitative observations and confirms that peptides P2, P4, and P5 induce significant alterations in cell morphology and actin cytoskeleton organization in both melanoma cell lines.

**Figure 9:**
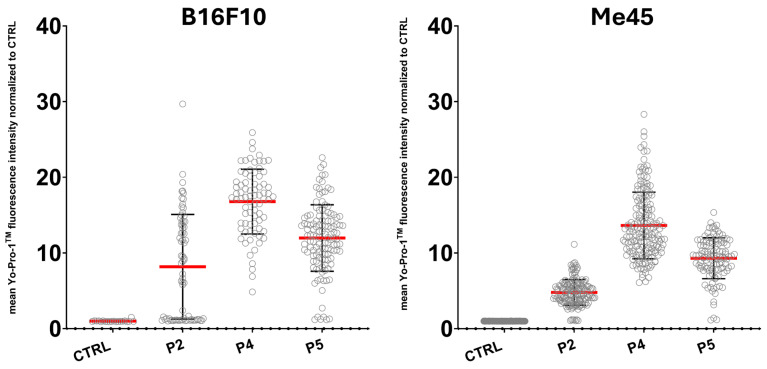
Quantification of Yo-Pro-1^TM^ fluorescence intensity in B16F10 and Me45 cells following treatment with peptides: P2 (P2; 1 mg/mL), P4 (P4; 0.25 mg/mL), and peptide P5 (P5; 1 mg/mL) for 1 h. The mean fluorescence intensity of Yo-Pro-1^TM^ was measured for individual cells and normalized to the untreated control (CTRL). Each gray circle represents a single cell measurement (≥70 cells per condition) and the red line indicates the mean fluorescence intensity across all measured cells for each condition.

**Figure 10:**
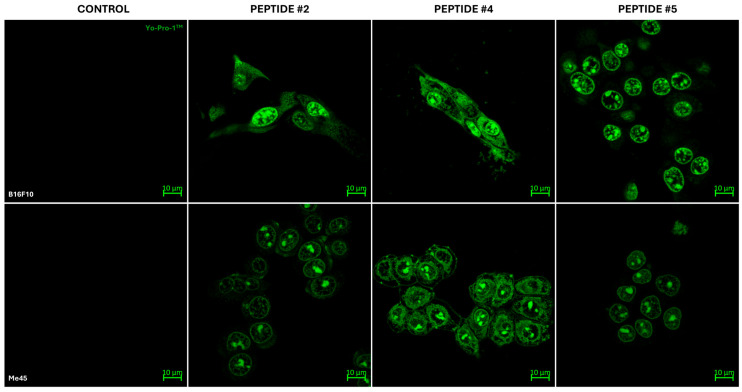
Yo-Pro-1^TM^ uptake in B16F10 and Me45 cells after 1 h incubation with peptides P2 (1 mg/mL), P4 (0.25 mg/mL), and P5 (1 mg/mL). Cells were stained with Yo-Pro-1^TM^ to assess membrane permeability and imaged using a confocal microscope with a 63× oil immersion objective. Representative images show differences in Yo-Pro-1^TM^ fluorescence depending on peptide treatment. Scale bar = 10 µm.

Cell membrane permeability was assessed in B16F10 and Me45 cell lines using Yo-Pro-1™ staining following 1 h of incubation with peptides P2, P4, or P5. Control cells (CTRL) were incubated in culture medium without peptide supplementation. The quantitative analysis is presented in Figure 9, while representative images are shown in Figure 10.

Control cells of both lines exhibited minimal dye signal, indicating low basal membrane permeability (Figure 9). In contrast, peptide-treated cells displayed increased fluorescence intensity, predominantly localized within the nuclear region, consistent with enhanced dye uptake.

In B16F10 cells, incubation with each of the tested peptides resulted in increased fluorescence intensity compared with the control. Quantitative analysis demonstrated an elevated-mean, normalized Yo-Pro-1™ fluorescence intensity under all experimental conditions, with the highest values observed following treatment with peptide P4 (an approximately 16-fold increase relative to CTRL). An increased but more variable signal was also observed following exposure to peptide P2 (approximately 8-fold increase relative to CTRL) and peptide P5 (approximately 12-fold increase relative to CTRL). These findings indicate increased membrane permeability following peptide exposure.

Similarly, in Me45 cells, an increase in fluorescence intensity was observed following peptide treatment compared with the control condition. Quantitative measurements revealed elevated normalized Yo-Pro-1™ signal across all analyzed conditions, with the most pronounced increase observed following exposure to peptide P4 (approximately 14-fold increase relative to CTRL). Peptides P2 and P5 also induced increased signal intensity, showing approximately 5-fold and 9.5-fold increases relative to control, respectively.

Collectively, these results indicate that short-term exposure to the investigated peptides influences the cellular permeability in both cell lines.

## 3. Discussion

**Figure 11:**
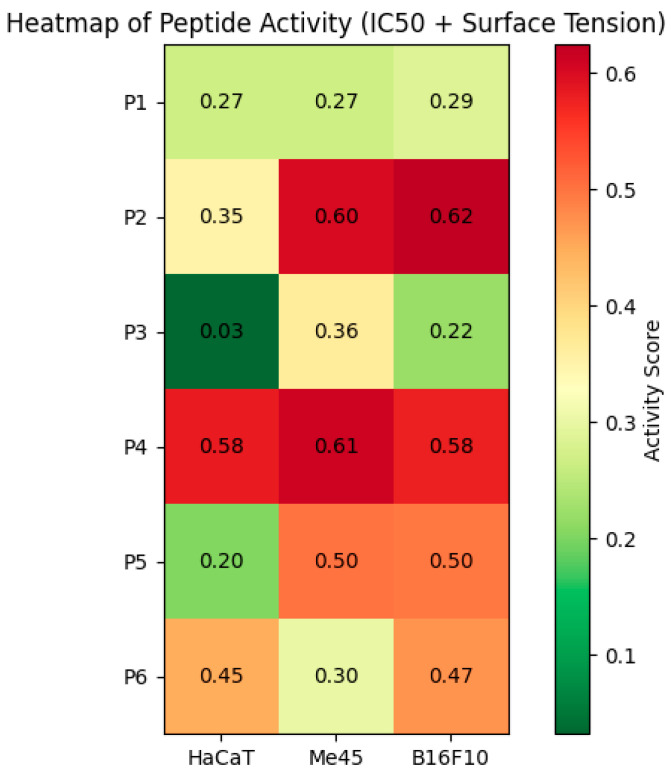
Heatmap of peptide activity, integrating IC_50_ cytotoxicity and surface tension (amphipathicity): combined activity scores were calculated by normalizing IC_50_ (inverted so lower values indicate higher activity) and surface tension to a 0–1 scale and averaging them to ensure reproducibility. Red regions indicate high activity (low IC_50_ + low surface tension → strong cytotoxicity and high amphipathicity), while green regions indicate low activity (high IC_50_ + high surface tension → weak cytotoxicity or weak amphipathicity).

The peptides P1–P6 demonstrated sustained chemical stability in NaCl solution, as evidenced by unchanged UV absorbance, confirming their suitability for subsequent in vitro cellular studies. The osmolality of physiological fluids, such as plasma, is typically around 275–300 mOsm/kg. The osmolalities of peptides P1–P6 range from 274 to 315 mOsm/kg, which is close to physiological conditions, thereby minimizing the risk of toxicity to healthy cells [42]. Notably, the higher osmotic pressure observed for P5 may induce osmotic stress in melanoma cells, potentially destabilizing the cell membrane. P2 shows a significant reduction in surface tension at concentrations of 0.0214–0.0641 mmol/L (33–34 mN/m), suggesting a strong ability to adsorb to surfaces and interact with lipids. P5 also reduces surface tension at intermediate concentrations (50–60 mN/m). The remaining peptides (P1, P3, P4, P6) showed less pronounced or more irregular changes in surface tension. Lowering the local surface tension may contribute to cell membrane disruption and increased permeability, potentially promoting pore formation [43]. The heatmap showing the combined activity of six cationic amphipathic peptides (P1–P6) across three cell lines (HaCaT, Me45, B16F10) is presented in Figure 11.

The activity score integrated cytotoxicity (IC_50_) and amphipathicity (surface tension). The data confirmed that P2 and P5 exhibited strong selective cytotoxicity against cancer cells while sparing healthy HaCaT cells, whereas P4 was non-selective and highly cytotoxic to all tested cell lines, in agreement with previous findings that cationic amphipathic peptides can display selective anticancer activity due to differential membrane interactions with cancer versus normal cells [44].

Sequence analysis of the P1–P6 peptides revealed differences in terms of charge, hydrophobic amino acid content, and possible modifications (e.g., C_12_ alkyl groups in P4). These factors are crucial for their behavior in solution and for interaction with biological membranes. The high positive charge facilitates electrostatic interaction with negatively charged cancer cell membranes, which often contain more phosphatidylserine and acidic glycolipids than healthy cell membranes [45,46]. Tryptophan stabilizes interactions with membranes through π–π coupling and interactions with aromatic lipids [47]. P2 and P5 are particularly rich in tryptophan, which explains their strong interaction with cancer cell membranes and high cytotoxic efficacy after 72 h. P4, on the other hand, despite its lower Trp content, has long C_12_ chains, making it very hydrophobic and leading to non-selective membrane damage. P4, with the lowest charge and highest hydrophobicity, had the lowest surface tension (44–53 mN/m) and a clearly defined CMC of 0.8511 mmol/L, indicating the ability to form micelles. The presence of tryptophan (Trp) and lysine (Lys) in the appropriate spatial conformation (amphipathicity) is crucial for the activity of the P5 peptide. Tryptophan acts as an “anchor,” stabilizing the peptide in the membrane interface (between the hydrophilic head and the hydrophobic lipid tail). At the same time, the positively charged lysine residues (+4 charge) provide a strong electrostatic attraction to the negatively charged cancer cell membranes. This specific structure is likely the reason for the peptide’s high selectivity. Peptide P3 contains arginine, which binds strongly to phosphate groups. Despite this, this peptide lacks a distinct amphipathic structure. It is characterized by greater flexibility and less hydrophobicity than peptide P2. This results in weak and non-targeted interactions with membranes, explaining its lack of selectivity and weak cytotoxicity (a significant effect is observed only at a very high concentration of 2 mg/mL). P6 has a concentrated, highly hydrophobic segment (WWW) at one end and a highly charged segment (KKKK) at the other. Concentrated tryptophans can lead to overly rapid and uncontrolled insertion into the membrane (not only the tumor membrane but any membrane it encounters), leading to non-selective lysis. P5 has a +4 charge that provides attraction, but the lack of strong, concentrated “anchoring” by tryptophans makes the interactions weak and requires very high concentrations (1.5 mg/mL) to elicit cytotoxic effects. The initial attraction of the peptides to the negatively charged POPG is driven by favorable electrostatic interactions between lysine residues and the lipid groups. The final stabilization of the complexes, however, is primarily driven by hydrophobic interactions, mainly involving tryptophan residues, reflecting the dominant entropic nature of the process [48]. Isothermal titration calorimetry measurements showed that the binding of peptides P2 and P5 to negatively charged POPG liposomes, although exothermic, is primarily entropically driven. The predominant entropic contribution (|Δ*H*| < |TΔ*S*|) may indicate the dominant importance of hydrophobic interactions in the stabilization of peptide–lipid complexes. These observations correlate with spectrofluorometric data: after the addition of liposomes, an increase in fluorescence intensity and a shift in the tryptophan emission maximum (by approximately 6 nm) were observed, confirming their insertion into the membrane and a key role in hydrophobic interactions [49,50,51].

Qualitative and quantitative analysis revealed that exposure of B16F10 and Me45 cells to peptides P2, P4, and P5 induced significant reorganization of the actin cytoskeleton, including weakening of stress fibers, reduced cell spreading, and a more diffuse F-actin signal, which persisted at both 24 h and 72 h and correlated with increased plasma membrane permeability, as measured by Yo-Pro-1™ uptake. These cytoskeletal disturbances are consistent with the literature describing the modulation of dynamic F-actin filaments as a key determinant of cellular responses to therapeutic agents and a regulator of cell mechanics, migration, and adhesion, processes critical for tumor progression and considered promising therapeutic targets in oncology [52,53]. Furthermore, membrane-active and amphipathic peptides are recognized as promising therapeutic tools due to their ability to selectively interact with cancer cell membranes, causing membrane destabilization and secondary cytoskeletal alterations, ultimately leading to apoptosis or reduced proliferation [54]. In light of these findings, the observed F-actin reorganization and increased membrane permeability suggest that the mechanism of action of P2 and P5 involves both direct membrane destabilization and secondary cytoskeletal disruption, potentially limiting adhesion, migration, and viability of cancer cells, whereas the more hydrophobic and less selective peptide P4 induces similar cytoskeletal effects independently of cancer cell specificity.

## 4. Materials and Methods

### 4.1. Synthesis and Characterization of the Peptide

Peptides P1–P6 were synthesized, purified, and structurally characterized according to the procedures described in our previous publication [32]. Their purity and identity were confirmed by high-performance liquid chromatography (HPLC, Nexera, Shimadzu, Tokyo, Japan) and mass spectrometry (MS, Christ, Hannover, Germany) [32].

### 4.2. Peptide Stability Study in NaCl

Peptide solutions P1–P6 were prepared at a concentration of 0.125 mg/mL using 0.9% NaCl (B. Braun Melsungen, Germany) for infusion. The UV/vis spectrum of each solution was measured in the range of 190–1100 nm to determine the absorbance maximum using a HaloDB-20 double-beam UV/vis spectrophotometer (Dynamica GmbH, Dietikon, Switzerland). The absorbance was then measured at the maximum wavelength for the given solution at time points 24 h, 96 h, 144 h, and 168 h. Each measurement was repeated three times.

### 4.3. Osmotic Pressure

Osmotic pressure of peptide solutions (P1–P6) was determined at concentrations of 0.125, 0.100, and 0.0625 mg/mL in 0.9% NaCl (B. Braun Melsungen, Germany). Measurements were performed using an. For each determination, 100 µL of the peptide solution was introduced into the measurement chamber. The instrument was calibrated using a NaCl standard, with a freezing point of –1.859 °C.

### 4.4. Surface Tension

Due to the limited availability of peptides P1–P6, the surface tension of aqueous peptide solutions was measured using a Langmuir–Wilhelmy trough (Kibron Microtrough X, Helsinki, Finland) equipped with FilmwareX 4.0 software. The setup included a platinum Wilhelmy plate with a mass of 48.2 mg and a diameter of 0.5 mm, ensuring a negligible contact angle during measurements.

To prevent contamination, the platinum plate was sequentially rinsed with methanol and distilled water before each measurement, followed by flame cleaning. The trough was placed on an anti-vibration table to minimize disturbances. All measurements were performed in triplicate. A Krüss thermostat (Hamburg, Germany) was used to maintain a constant temperature of 25 °C throughout the experiments.

The critical micelle concentration (CMC) was determined using the surface tension method. Surface tension values were measured for peptide solutions at different concentrations and plotted as a function of the logarithm of peptide concentration. The CMC value was determined from the breakpoint of the obtained curve. Linear regressions were applied to the regions before and after the change in slope, and the intersection of these two lines corresponded to the CMC value [55,56]. Surface tension was measured using a tensiometer equipped with a cylindrical Wilhelmy plate immersed in the aqueous phase. The Wilhelmy method is based on measuring the force acting on a thin plate partially immersed at the air–liquid interface. This force depends on the surface tension of the liquid, the wetted perimeter of the plate, and the contact angle between the plate and the liquid surface. The relationship can be expressed as

$$F={⍴}_{g}g{\pi r}^{2}l+2\gamma \left(\pi r\right)cos\theta -{⍴}_{l}{\pi r}^{2}h$$

F—net force [N], ⍴g—wire density [kg/m3], ⍴l—the density of the subphase [kg/m3], g—gravitational constant [n/kg], r—wire radius [m], l—wire length [m], h—insertion depth of the wire [m], γ—the surface tension of the liquid [mN/m], θ—contact angle [57].

For complete wetting, the contact angle was assumed to be 0° (cos θ = 1). The contribution of the gas phase was neglected in the calculations because its density was negligible compared to that of the liquid. The difference in force between pure water and film-covered water is

$$F=2\pi r(\gamma -\gamma^{i})$$

Here, $\gamma -\gamma^{i}$ represents the surface pressure of the film [58].

Analysis of variance (ANOVA) was performed at a confidence level of 95% using Statistica (data analysis software system), version 13, with the Tukey test [59].

### 4.5. Cell Culture

Human melanoma cells Me45, murine melanoma cells B16F10, and immortalized human keratinocytes HaCaT were obtained from the ATCC^®^ (American Type Culture Collection, Manassas, VA, USA). Cells were cultured in Dulbecco’s Modified Eagle Medium (DMEM; L0104–500, Biowest, Nuaillé France) supplemented with 10% fetal bovine serum (FBS; E5050-02, EURx, Poland) and a 1% penicillin/streptomycin solution (100 U/mL penicillin and 100 µg/mL streptomycin; A5955-100ML, Sigma-Aldrich, Steinheim, Germany). Cells were maintained under standard culture conditions (37 °C, 5% CO_2_, 95% humidity). Cultures were propagated in T-25/T-75 culture flasks (83.3910.302 or 83.3911.302; Sarstedt, Nümbrecht, Germany) and subcultured upon reaching 70–80% confluence. For cell detachment, TrypLE™ Express Enzyme (12563011; Thermo Fisher Scientific Inc., Waltham, MA, USA) was used.

### 4.6. Cell Viability Assay—MTT

Cell viability was assessed using the MTT assay (3-(4,5-dimethyl-2-thiazolyl)-2,5-diphenyl tetrazolium bromide, Sigma-Aldrich, Steinheim, Germany). Me45, B16F10, and HaCaT cells were suspended in culture medium and seeded into 96-well plates at a density of 4 × 10^4^ cells/well in 200 µL. After 12 h of incubation under standard conditions (37 °C, 5% CO_2_, 95% humidity) to allow cell adhesion, cells were treated with peptide solutions P1–P6 at the following concentration ranges: 0.023–1.5 mg/mL for P1 and 0.03–2 mg/mL for P2–P6. Peptide concentrations were adjusted individually based on physicochemical properties and preliminary cytotoxicity screening to ensure informative dose–response evaluation. Incubations were carried out for 24 or 72 h under the same culture conditions.

Following incubation, the culture medium was aspirated and 100 µL of MTT solution (0.5 mg/mL in culture medium) was added to each well, followed by 2 h of incubation (37 °C, 5% CO_2_, 95% humidity). The resulting formazan crystals were solubilized in 100 µL of acidified isopropanol (38% HCl in 99.7% isopropanol). Absorbance was measured spectrophotometrically at 570 nm with background correction at 630 nm using a GloMax^®^ Discover microplate reader (Promega, Madison, WI, USA).

Cell viability was expressed as a percentage relative to untreated control cells (CTRL). All experiments were performed in at least two independent biological replicates, each with three technical replicates per condition. Values above 100% in the MTT assay reflected increased mitochondrial/metabolic activity relative to the untreated control and did not necessarily indicate a proportional increase in cell number [60].

Statistical analyses were performed using GraphPad Prism 8 (GraphPad Software, La Jolla, CA, USA). Data are presented as the mean ± standard deviation (SD). Prior to statistical analysis, the normality of data distribution was evaluated using the Shapiro–Wilk test. Differences between groups were analyzed using two-way analysis of variance (two-way ANOVA) followed by Dunnett’s post hoc test for multiple comparisons against the control group. Differences were considered statistically significant at *p* < 0.05.

### 4.7. Determination of IC_50_ Values and Selectivity Index (SI) Values

IC_50_ values were calculated from the dose–response curves obtained in the MTT assay. Nonlinear regression analysis was performed using GraphPad Prism 10 (four-parameter logistic model, variable slope). For peptides that did not reduce cell viability below 50% within the tested concentration range, IC_50_ values were reported as “> maximal tested concentration”. All analyses were based on mean viability values from three independent experiments.

To evaluate the selectivity of the tested peptides toward melanoma cells relative to normal keratinocytes, the selectivity index (SI) was calculated based on the IC_50_ values obtained from the MTT assay. The SI was determined according to the following formula:

$$SI=\frac{{IC}_{50}HaCaT}{{IC}_{50}Me45/B16F10}$$

where IC_50_ HaCaT represents the IC_50_ value for normal human keratinocytes (HaCaT) and IC_50_ cancer represents the IC_50_ value for melanoma cell lines (Me45 or B16F10).

When the IC_50_ value for HaCaT cells exceeded the highest tested concentration, a minimal selectivity index (SI_min_) was calculated using the highest tested concentration value as the numerator. If IC_50_ values were not reached within the tested concentration range in both compared cell lines, the selectivity index was not determined. Higher SI values indicated greater selectivity of the compound toward melanoma cells compared with normal keratinocytes.

### 4.8. Liposome Preparation

Liposomes were prepared by hydration of a dried lipid film. Briefly, the required amount of 1-palmitoyl-2-oleoyl-sn-glycero-3-phospho-(1′-rac-glycerol) (sodium salt) (POPG) was dissolved in chloroform, and the solvent was removed under a stream of nitrogen. The samples were additionally lyophilized overnight to ensure complete removal of residual chloroform. The dry lipid film was then hydrated with phosphate-buffered saline (PBS; 0.01 M phosphate buffer, 0.0027 M KCl, 0.137 M NaCl, pH 7.4) to a final lipid concentration of 1.3 mM and gently shaken for 2 h at 318 K to form multilamellar vesicles (MLVs). Next, the lipid suspensions were subjected to five freeze–thaw cycles (freezing in liquid nitrogen and thawing at 318 K), followed by a final thawing step at room temperature to reduce lamellarity and MLV size. Finally, the lipid suspension was extruded through polycarbonate membranes with a 100 nm pore size using an Avanti mini-extruder (Avanti Polar Lipids, Inc., Alabaster, AL, USA.) to obtain large unilamellar vesicles (LUVs) of ~100 nm in diameter. All the reagents were prepared freshly before measurements.

### 4.9. Isothermal Titration Calorimetry (ITC)

Isothermal titration calorimetry (ITC) experiments were performed at a temperature of 298.15 K using an Auto-ITC instrument (MicroCal, GE Healthcare, Northampton, MA, USA). The sample cell contained a buffered solution of P2 or P5 at a concentration of 0.05 mM. Titrations involved the addition of 1.3 mM buffered solution of POPG as the titrant. Each injection was conducted over 20 s, with 240 s pauses between injections to allow for equilibrium. The heat of dilution for the POPG was measured separately in control experiments and subsequently subtracted from the titration data. The stoichiometry and thermodynamic parameters (*K*_(ITC)_ and Δ*H*_(ITC)_) were directly determined from ITC measurements by fitting the isotherms using nonlinear least-squares methods to a model that assumed a single set of binding sites [61]. The standard thermodynamic equations, Δ*G*_(ITC)_ = −R*T*ln*K*_(ITC)_ = Δ*H*_(ITC)_ − *T*Δ*S*_(ITC)_, were employed to calculate the binding free energy, Δ*G*_(ITC)_, and the entropy change, Δ*S*_(ITC)_.

### 4.10. Steady-State Fluorescence Spectroscopy (SF)

Fluorescence emission spectra were recorded using the Cary Eclipse Varian (Agilent, Santa Clara, CA, USA) spectrofluorometer, equipped with a temperature controller and multicell holder, at 298.15 K using the stock solutions of the P2/P5 peptides (10 µM) and POPG (1.3 mM), which were prepared in 10 mM PBS buffer (pH 7.4). The excitation wavelength was 295 nm. The widths of the excitation and emission slits were set at 10 and 5 nm, respectively. In the absence and presence of increasing concentrations of POPG, up to 31.7 µM, the fluorescence emission spectra of the P2 and P5 peptides were recorded from 300 to 500 nm. For this purpose, ten additions of 5 µL stock solution of POPG (1.3 mM) were used to titrate 2 mL of each peptide at a fixed concentration equal to 10 µM. The mixture was shaken and the spectra were recorded after 3 min of each addition of POPG.

### 4.11. Membrane Permeability and F-Actin Staining

B16F10 and Me45 cells were seeded onto glass-bottom dishes (µ-Dish 35 mm, high Glass Bottom, ibidi, cat. no. 80807) and maintained under standard culture conditions (37 °C, 5% CO_2_). Cells were incubated with peptides P2 (1 mg/mL), P4 (0.25 mg/mL), or P5 (1 mg/mL) for 1 h for membrane permeability assessment or for 24 and 72 h for actin cytoskeleton visualization. Untreated cells served as controls (CTRL).

To evaluate membrane permeability, cells were incubated with the green fluorescent nucleic acid dye Yo-Pro™-1 (λ_exc 491 nm/λ_em 509 nm; Thermo Fisher Scientific Inc., Waltham, MA, USA; cat. no. Y3603) diluted 1:1000 in a culture medium, according to the manufacturer’s instructions. Following staining, cells were imaged immediately without fixation. For visualization of the actin cytoskeleton, cells were stained with CellMask™ Green Actin Tracking Stain (Thermo Fisher Scientific, cat. no. A57243), diluted 1:1000 in a culture medium, and incubated for 1 h at 37 °C in 5% CO_2_, according to the manufacturer’s protocol.

Fluorescence imaging was performed using a ZEISS LSM 980 laser scanning confocal microscope equipped with Airyscan 2 (Carl Zeiss, Oberkochen, Germany). Quantitative analysis of Yo-Pro™-1 fluorescence was performed using a 20× objective, while representative high-resolution images were acquired using a 63× oil immersion objective. All imaging and analyses were conducted using identical acquisition and processing parameters to ensure comparability between experimental conditions.

Quantitative analysis was performed by measuring the mean fluorescence intensity in individual cells (>70 cells per condition) using ZEN software (version 3.3, Carl Zeiss, Oberkochen, Germany). Fluorescence intensity values were normalized to the CTRL condition. Graphical representation of quantitative data (Figure 7) was generated using GraphPad Prism (version 10.1.1, GraphPad Software, La Jolla, California, USA). Representative fluorescence images are shown in Figure 8. Actin cytoskeleton organization was additionally evaluated qualitatively based on cell morphology and F-actin distribution, as presented in Figure 6.

## 5. Conclusions

P2 and P5 exhibited the most favorable properties for further development as therapeutic agents, showing strong and selective cytotoxicity against cancer cells with limited effects on normal cells. In contrast, peptide P4 was strongly cytotoxic at concentrations ≥0.5 mg/mL, rapidly reducing melanoma cell viability but also affecting HaCaT cells, and caused nearly complete loss of viability at higher concentrations, reflecting potent but non-selective membrane disruption likely related to micellization. P3 and P1 were moderately active and lacked full selectivity, while P6 was weakly active with an unfavorable toxicity profile. These results confirmed that the interplay of several physicochemical factors, including sequence, charge, spatial structure, osmotic pressure, and surface tension, are crucial for the activity of anticancer peptides. Moreover, the observed F-actin cytoskeletal reorganization and increased membrane permeability induced by P2 and P5 indicate that their cytotoxicity involves both direct membrane destabilization and secondary disruption of cytoskeletal integrity, contributing to reduced adhesion, migration, and survival of cancer cells.

## Figures and Tables

**Table 1:** Osmotic pressure values depending on the concentration. For each concentration, three measurement replicates were performed. Since all obtained values were identical, the results are presented as mean ± SD, with SD = 2.89 mOsm/kg.

Name	Structure	Net Charge	MW (g/mol)	Osmotic Pressure (mOsm/kg) *	Temperature (°C) *
P1	(WK)_2_-KWK-NH_2_	+5	1087.64	296, 293, 289	−0.549, −0.544, −0.536
P2	(WKWK)_2_-KWKWK-NH_2_	+8	2030.16	278, 277, 275	−0.516, −0.514, −0.510
P3	(WR)_2_-KWR-NH_2_	+5	1171.66	282, 280, 276	−0.523, −0.519, −0.512
P4	(C12)_2_-KKKK-NH_2_	+3	893.70	281, 278, 274	−0.522, −0.517, −0.509
P5	(KWK)_2_-KWWW-NH_2_	+4	1587.89	315, 302, 289	−0.585, −0.561, −0.536
P6	(KK)_2_-KWWW-NH_2_	+4	1215.73	280, 277, 275	−0.520, −0.514, −0.510

* Osmotic pressure (mOsm/kg) and temperature (°C) measured sequentially for concentrations of 0.125, 0.100, and 0.0625 mg/mL.

**Table 2:** IC_50_ values obtained from MTT viability assay for peptides P1–P6 following 24 h and 72 h treatment with HaCaT, Me45, and B16F10 cells.

	HaCaT		Me45		B16F10	
Time [Hours]	24	72	24	72	24	72
**P1**	*>1.5*	*>1.5*	*>1.5*	*>1.5*	1.374	0.7152
**P2**	*>2.0*	*>2.0*	*>2.0*	0.3907	*>2.0*	0.2385
**P3**	3.207	20.94	1.077	1.329	*>2.0*	*>2.0*
**P4**	0.3129	0.4093	0.1231	0.2077	0.3386	0.2558
**P5**	*>2.0*	*>2.0*	5.191	0.1083	*>2.0*	0.1319
**P6**	1.064	1.162	*>2.0*	*>2.0*	*>2.0*	0.9135

**Table 3:** Thermodynamic parameters of binding interactions of POPG with P2 and P5 in a PBS buffer (pH 7.4) at 298.15 K (standard deviation values in parentheses). *N*—stoichiometry, log*K*_(ITC)_—logarithm of the binding constant, Δ*G*_(ITC)_ [kcal mol^−1^]—Gibbs free energy, Δ*H*_(ITC)_ [kcal mol^−1^]—enthalpy, *T*Δ*S*_(ITC)_ [kcal mol^−1^]—entropic contribution.

ITC Data for POPG Complexes		
Parameter	P2	P5
*N*	1.55 (±0.06)	1.83 (±0.06)
log*K*_(ITC)_	6.15 (±0.33)	5.58 (±0.20)
Δ*G*_(ITC)_ [kcal mol^−1^]	−8.38 (±0.45)	−7.62 (±0.27)
Δ*H*_(ITC)_ [kcal mol^−1^]	−0.37 (±0.02)	−0.57 (±0.03)
*T*Δ*S*_(ITC)_ [kcal mol^−1^]	8.02	7.05

## Data Availability

The scientific data are available at Wroclaw Medical University, Department of Physical Chemistry and Biophysics.

## Supplementary Materials

The following supporting information can be downloaded at https://www.mdpi.com/article/10.3390/ijms27072952/s1.
