# Supplementary material for: Physicochemical Characteristics of Amphipathic Peptides and Their Cytotoxic Effects on Cancer and Normal Cell Lines
Source: Int J Mol Sci. 2026 Mar 24;27(7):2952. doi: 10.3390/ijms27072952 (PMC13073417; doi:10.3390/ijms27072952)
Supplement: Supplementary file 1 [file ijms-27-02952-s001.zip › ijms-4186629-supplementary.pdf]

Supplementary Materials

Table S1. Stability of peptides P1–P6 in NaCl solution (0.125 mg/ml) monitored by UV absorbance over time. For each concentration, three measurement replicates were performed. All obtained values were identical; therefore, the results are presented as mean ± SD (SD = 0.001 A).

Sample	Wavelength (nm)	24 h	96 h	144 h	168 h
**P1**	279	1.06	1.06	1.06	1.06
**P2**	278	2.64	2.64	2.64	2.64
**P3**	279	1.13	1.13	1.13	1.13
**P4**	204	1.38	1.38	1.38	1.38
**P5**	280	1.34	1.34	1.34	1.34
**P6**	280	1.20	1.20	1.20	1.20

Table S2. IC_{50} values together with Selectivity Index (SI) and minimal Selectivity Index (SImin) derived from MTT viability assay for peptides P1–P6 after 24 h and 72 h exposure of HaCaT, Me45, and B16F10 cells.

	HaCaT		Me45				B16F10			
	24 H	72 H	24 H		72 H		24 H		72 H	
	IC50	IC50	IC50	SI	IC50	SI	IC50	SI	IC50	SI
**P1**	*> 1.5*	*> 1.5*	*> 1.5*	*IC_{50} not reached in any tested cell line*	*> 1.5*	*IC_{50} not reached in any tested cell line*	*> 1.5*	*IC_{50} not reached in any tested cell line*	0,715	2,097
**P2**	*> 2.0*	*> 2.0*	*> 2.0*	*IC_{50} not reached in any tested cell line*	0,391	5,119	*> 2.0*	*IC_{50} not reached in any tested cell line*	0,239	8,386
**P3**	3,207	20,940	1,077	2,978	1,329	15,760	*> 2.0*	*potentially greater toxicity to normal cells*	*> 2.0*	*potentially greater toxicity to normal cells*
**P4**	0,3129	0,4093	0,1231	2,542	0,208	1,971	0,339	0,924	0,256	1,600
**P5**	*> 2.0*	*> 2.0*	5,191	*0,385 lack of selectivity towards cancer cells*	0,108	18,467	*> 2.0*	*IC_{50} not reached in any tested cell line*	0,132	15,163
**P6**	1,064	1,162	*> 2.0*	*potentially greater toxicity to normal cells*	*> 2.0*	*potentially greater toxicity to normal cells.*	*> 2.0*	*potentially greater toxicity to normal cells*	0,914	1,272
